# Genetic structure of the crown-of-thorns seastar in the Pacific Ocean, with focus on Guam

**DOI:** 10.7717/peerj.1970

**Published:** 2016-05-05

**Authors:** Sergio Tusso, Kerstin Morcinek, Catherine Vogler, Peter J. Schupp, Ciemon F. Caballes, Sergio Vargas, Gert Wörheide

**Affiliations:** 1 Department of Earth and Environmental Sciences, Palaeontology & Geobiology, Ludwig-Maximilians-Universität München, München, Germany; 2 Department of Anatomy (Neuroanatomy), University of Cologne, Köln, Germany; 3 Environment Department, Pöyry Switzerland Ltd., Zurich, Switzerland; 4 Environmental Biochemistry, Carl-von-Ossietzky University Oldenburg, ICBM-Terramare, Wilhelmshaven, Germany; 5 ARC Centre of Excellence for Coral Reef Studies, James Cook University, Townsville, Queensland, Australia; 6 SNSB-Bavarian State Collections of Palaeontology and Geology, München, Germany; 7 GeoBio-Center, Ludwig-Maximilians-Universität München, München, Germany

**Keywords:** *Acanthaster ‘planci’*, Microsatellites, Pacific, Genetic structure, Crown-of-thorns seastar, COTS

## Abstract

Population outbreaks of the corallivorous crown-of-thorns seastar (COTS), *Acanthaster ‘planci’ L.*, are among the most important biological disturbances of tropical coral reefs. Over the past 50 years, several devastating outbreaks have been documented around Guam, an island in the western Pacific Ocean. Previous analyses have shown that in the Pacific Ocean, COTS larval dispersal may be geographically restricted to certain regions. Here, we assess the genetic structure of Pacific COTS populations and compared samples from around Guam with a number of distant localities in the Pacific Ocean, and focused on determining the degree of genetic structure among populations previously considered to be isolated. Using microsatellites, we document substantial genetic structure between 14 localities from different geographical regions in the Pacific Ocean. Populations from the 14 locations sampled were found to be structured in three significantly differentiated groups: (1) all locations immediately around Guam, as well as Kingman Reef and Swains Island; (2) Japan, Philippines, GBR and Vanuatu; and (3) Johnston Atoll, which was significantly different from all other localities. The lack of genetic differentiation between Guam and extremely distant populations from Kingman Reef and Swains Island suggests potential long-distance dispersal of COTS in the Pacific.

## Introduction

The crown-of-thorns seastar (COTS), *Acanthaster ‘planci,’* is a specialised coral predator and one of the most important biological threats to coral reefs throughout the Indo-Pacific (Pratchett et al., 2014). It has a complicated taxonomic history; although initially considered a single widespread Indo-Pacific species (reviewed in Haszprunar & Spies, 2014), recent molecular data suggests that *Acanthaster ‘planci’* is a species complex consisting of at least four different species (Vogler et al., 2008), all of them showing internal phylogeographic structure, and one of which is largely restricted to the Pacific (Vogler et al., 2012; Vogler et al., 2013). Since formal description of these species is still pending, we refer to the Pacific species as *Acanthaster ‘planci’* or COTS hereafter. COTS predatory behaviour has resulted high levels of coral mortality. For example, massive outbreaks on the northwest coast of Guam in the late 1960s reduced coral cover down to <10% (Chesher, 1969) and coral species richness decreased from 146 to 96 one year after the outbreaks (Randall, 1973). As a consequence, the community structure of affected coral reefs have often been significantly altered, promoting algal colonization and affecting fish population dynamics (Pratchett et al., 2014).

Although frequently studied, the origin, development and causes of COTS outbreaks remain largely unclear (Birkeland & Lucas, 1990; Kayal et al., 2012; Pratchett et al., 2014; Vogler et al., 2013; Yasuda et al., 2009). Different authors (i.e., Benzie, 1999a; Brodie et al., 2005; Gérard et al., 2008; Scheltema, 1986) have highlighted the importance of larval survival and dispersal in explaining COTS outbreaks. A single female COTS can produce more than 60 million eggs per spawning season (Conand, 1984) and this can result in more than 10 million fertilised eggs per year per mature female (Benzie et al., 1994). Therefore, a small increase in the survival rate of the COTS larvae could lead to a rapid increase in population size (Brodie et al., 2005) and geographic spread, considering a planktonic larval duration (PLD) ranging between 9 and 42 days (reviewed in Caballes & Pratchett, 2014). Different variables, such as an enhanced food supply (Brodie et al., 2005; Fabricius, Okaji & De’ath, 2010), reduced predation pressure due to overfishing (Sweatman, 2008), and changes in diverse environmental variables (e.g., sea surface temperature or rainfall; Black et al., 1995; Brodie et al., 2005; Glynn, 1985; Houk, Bograd & Van Woesik, 2007) have been postulated to increase larval or adult survival and promote COTS outbreaks. Additional explanatory hypotheses on the local origin of outbreaks are given by changes in behaviour or survivorship of post-settlement individuals, e.g., a decrease in predation; (Endean, 1969), the movement of adults between reefs (Talbot & Talbot, 1971), adult aggregation (Dana, Newman & Fager, 1972), or outbreak cycles controlled by increase in pathogen transmission under high densities (reviewed in Pratchett et al., 2014).

Considering the microscopic size of COTS larvae, analyses of its population dynamics to understand the structure and origin of outbreaks have focused on indirect molecular methods. This approach is grounded on the assumption that organisms with short planktonic stages and low spatial dispersal capabilities have higher population genetic structure (resulting from lower levels of gene flow) than those with longer planktonic stages, which are thought to have higher levels of gene flow and reduced population genetic structure. A correlation between the potential for migration and genetic structure has been observed in different marine groups, including other seastars (e.g., *Linckia laevigata*; Benzie, 1999b), and different species of corals (e.g. Ayre & Hughes, 2000; Nishikawa, Katoh & Sakai, 2003). In the case of *A. ‘planci’*, a species with a long-lived planktonic larval stage (Birkeland & Lucas, 1990; Caballes & Pratchett, 2014), reduced genetic structure and high migration rates have been assumed (Benzie, 1999a).

Initial studies using allozymes to investigate COTS population genetics seemed to provide evidence of strong gene flow and lack of genetic structure (Benzie, 1999a; Benzie & Stoddart, 1992; Nash, Goddard & Lucas, 1988; Nishida & Lucas, 1988). However, more recent analyses using different molecular markers have pointed towards a different scenario. Using the mitochondrial control region (Timmers et al., 2012; Vogler et al., 2012; Vogler et al., 2013), internal genetic differentiation was observed within at least three of the four different clades (species) of *A.‘planci’* (i.e., the Pacific, the Northern and the Southern Indian Ocean clades) proposed by Vogler et al. (2008). Vogler et al. (2013) found support for at least four genetic groups in the Pacific Ocean and Timmers et al. (2012) discovered reduced gene flow among regions and archipelagos and significant genetic differentiation between COTS populations from the Central Pacific Ocean. Thus COTS dispersal seems to be limited to smaller geographic areas, for instance within the Great Barrier Reef (GBR) (Benzie & Stoddart, 1992; Benzie & Wakeford, 1997), in the Ryukyus Islands (Yasuda et al., 2009) and along the Hawaiian Archipelago (Timmers et al., 2011).

Although mitochondrial markers show genetic differentiation between populations that have large scale geographic structure (Timmers et al., 2012; Vogler et al., 2013), these markers have not allowed differentiation between historical evolutionary migration and contemporary gene flow. For example, Timmers et al. (2012) showed that there are shared mitochondrial haplotypes between the South Central and Northwest Pacific and that their haplotypes do not strictly cluster according to geographic region. This pattern was interpreted as either recent gene flow, the retention of ancestral polymorphisms or ancestral gene flow (Timmers et al., 2012). Similarly, Vogler et al. (2013) found a large geographic cluster of Western Pacific localities, with shared haplotypes in the whole range from the GBR to the Philippines. However, microsatellite data (Yasuda et al., 2009) show that significant genetic differentiation in this region is more pronounced, with patterns of isolation by distance and significant pairwise ${F}_{\mathrm{ST}}$ values (fixation index) between several localities indicating intra-cluster genetic differentiation.

The differentiation between contemporary gene-flow patterns and evolutionary history is of importance for conservation biology as highlighted by several authors (e.g., Peijnenburg et al., 2006; Selkoe & Toonen, 2006; Eytan & Hellberg, 2010; Van der Meer et al., 2012). The mutation rate of mitochondrial DNA is known to be suitable to resolve taxonomic uncertainties and historical biogeographical events, but it may not be suitable to infer contemporary migration events (Wan et al., 2004). Microsatellites, which evolve up to 100 times faster than mitochondrial DNA, provide enough variance for inferring patterns of gene flow and contemporary genetic structure (Wan et al., 2004), especially at smaller geographical scales. Despite their many advantages, there are only two studies using microsatellites from COTS and they are mainly concerned in the connectivity patterns among Western Pacific populations (Yasuda et al., 2009) and locally at the Society Islands, French Polynesia (Yasuda et al., 2014). The applicability of microsatellites to investigate the relatedness of Pacific COTS populations and their genetic structure over larger geographic distances has not been tested yet.

This study aims to investigate the contemporary genetic structure of the Pacific crown-of-thorns seastar species using microsatellites and to test for isolation among distant geographical regions previously identified as a cohesive genetic unit by mitochondrial DNA. We especially focus on the genetic structure of populations around Guam, where recent COTS outbreaks have been observed. We compared samples from around Guam with a number of distant localities in the Pacific Ocean, and focused on determining the degree of genetic structure among populations previously considered to be isolated (i.e., Johnston Atoll, Kingman Reef, Swains Island, Japan, the Great Barrier Reef , Vanuatu, Moorea, and Philippines).

**Figure 1:**
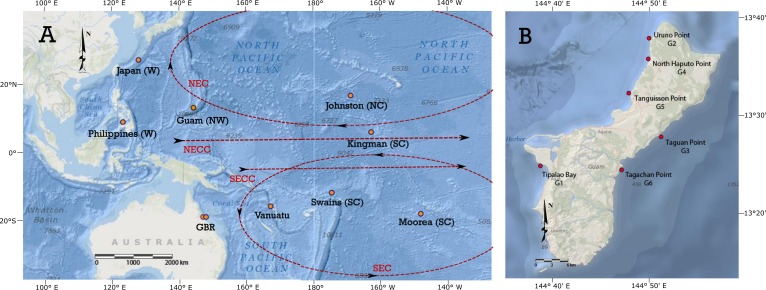
*Acanthaster ‘planci’* localities sampled in the Pacific Ocean and around Guam. (A) *Acanthaster ‘planci’* [i]localities sampled in the Pacific Ocean. Localities are coded by geographical regions: west Pacific (W), north-west Pacific (NW), north-central Pacific (NC), south-central Pacific (SC). GBR represents the Great Barrier Reef. Current paths are presented in dashed line: North Equatorial Countercurrent (NECC), North Equatorial Current (NEC), South Equatorial Countercurrent (SECC), and South Equatorial Current (SEC). (B) Details of sampling locations around Guam. Maps are taken and modified from www.arcgis.com. Source: Esri, GEBCO, DeLorme, NaturalVue—Esri, GEBCO, IHO-IOC GEBCO, DeLorme, NGS.

## Materials and Methods

### Sampling

Guam is the largest and most southern island of the Marianas archipelago. It is located in the Western Pacific Ocean within Micronesia (Fig. 1). The impact of *A. ‘planci’* on this island has been reported since the early 1970’s (Gawel, 1999), and surveys from 2003 to 2007 found numerous outbreaks in different coral reefs around the island and detected an increase in outbreak intensity in each subsequent expedition (Burdick et al., 2008). A total of 172 tube feet samples were collected by SCUBA diving and snorkelling from six reef locations around Guam in 2006 (Table 1). No permits were required for COTS collection as COTS is not a protected species and collections were done outside protected areas. Five of the sampled localities around Guam had densities of more than 150 COTS per hectare, with Tanguisson Reef (G5) having the highest density of 522 COTS per hectare. Only one locality, Taguan Point (G3), was considered to be a non-outbreak population (<30 COTS per hectare). Another 102 COTS tube feet or pyloric caeca samples were collected from nine reef locations in the Pacific (Fig. 1; Table 1). The choice of sampling locations outside Guam was based on previous studies using sequences of the mitochondrial control region and designed to include localities from the most distinguishable genetic groups in the Pacific (Vogler et al., 2013): West, North-Central, North-West and South-Central Pacific (Fig. 1). Since the goal of this study was to evaluate the genetic structure between distant localities, the sampling included islands ranging in distance from over 2,000 km between Guam and Japan to over 5,000 km between Guam and Kingman Reef. Although genetic connectivity has not been reported between some of the sampled localities (e.g., Johnston Atoll and Guam) and oceanic currents predict isolation, these localities were also included here to characterise the variance of the markers used, control for possible homoplasy or as an internal methodological control, assuming that those populations should be genetically highly differentiated.

**Table 1:** Summary information of sampled localities. Code of locality, collection year, number of samples (N), number of alleles (Na), allelic richness (Ar), observed heterozygosity (Ho), expected heterozygosity (He), and inbreeding coefficient (Fis) are given.

Sample location	Code	Collection year	N	Na	Ar	Ho	He	Fis	HW-test *P*-value
Tipalao, Guam	G1	2006	20	6.9	5.01	0.6708	0.6896	0.028	0.593
Uruno, Guam	G2	2006	30	8.0	5.42	0.6745	0.7361	0.085	0.022
Taguan, Guam	G3	2006	32	7.3	5.04	0.6987	0.7047	0.009	0.171
North Haputo, Guam	G4	2006	30	7.5	5.14	0.6533	0.7008	0.069	0.015
Tanguisson, Guam	G5	2006	30	7.3	5.00	0.6900	0.7176	0.039	0.185
Tagachan, Guam	G6	2006	30	7.3	5.07	0.6467	0.7055	0.085	0.184
Johnston Atoll	J	2006	9	3.1	2.85	0.3333	0.4575	0.284	0.053
Kingman reef	K	2006	20	5.5	4.37	0.5833	0.6704	0.133	0.004
Swains	S	2006	10	5.4	4.87	0.6643	0.7228	0.085	0.042
Japan	Ja		18	6.8	5.02	0.4923	0.6869	0.290	0.000
GBR	GBR	1999	19	7.0	5.22	0.3458	0.7442	0.542	0.000
Vanuatu	V		11	5.0	4.52	0.3685	0.7101	0.494	0.000
Moorea	M	2006	5	3.3		0.3556	0.6173	0.617	0.006
Phillipines	P		11	5.4	4.70	0.2795	0.6558	0.588	0.000

The samples were stored in ethanol 80% or DMSO buffer at −80 °C. A MagAttract 95 DNA Plant Core Kit (Qiagen) was used to extract total DNA from tube feet and pyloric caeca samples, following the protocol recommended by the manufacturer. As a preliminary step, the tissue was ground after freezing in liquid nitrogen, and incubated for 1 h at 35 °C in RLT lysis buffer (Qiagen). In the case of tube feet, DNA was extracted using the DNeasy Tissue Kit (Qiagen), according to the protocol recommended by the manufacturer.

All samples were genotyped using a set of 13 microsatellites previously identified for *A. ‘planci’* (Yasuda et al., 2006; Yasuda et al., 2007). The set included the loci Yukina01, Yukina05, Yukina06, Yukina08, Maki01, Maki03, Tama01 and Hisayo01 from Yasuda et al. (2006) and Aya02, Maki12, Maki11, Tama11 and AyU03 from Yasuda et al. (2007). Standard three-step PCR reactions were conducted for each locus in a final volume of 12.5 µl of GoTaq Flexi Buffer^®^ 1x, MgCl_2_ 3 mM, dNTPs 0.4 mM, primers forward and reverse 0.2 µM, BSA 0.08 mg/ml and 0.5u of GoTaq^®^ polimerase (Promega) with 1 µl of DNA template (around 20 ng of DNA). For fragment length analysis, the 5′ end of the forward primers used in the PCR were labelled with a fluorescent dye (HEX, 6-FAM or BoTMR). The PCR cycling conditions were as follows: 10 min at 94 °C, 38 cycles of 30 s at 94 °C, 30 s at 56–60 °C (primer-specific annealing temperature), and 1min at 72 °C, and a final elongation of 5 min at 72 °C.

PCR products were mixed for genotyping in 3 different co-loading reactions as follows: co-loading 1 included loci Yukina01, Yukina05, Yukina06 and Yukina08; co-loading 2 included Maki01, Maki03, Tama01 and Hisayo01; and co-loading 3 included Aya02, Maki12, Maki11, Tama11 and AyU03. Samples were analysed on an ABI 3730 48 capillary sequencer (Applied Biosystems) using the dye set D and G5 and 400HD ROX size standard at the Sequencing Service of the Department of Biology at the Ludwig-Maximilians-Universität in Munich (Germany). The software GeneMapper v.4.1 was used to call allele sizes. The raw genotype data is given in Table S1.

### Data analysis

The Markov chain algorithm implemented in the software GENEPOP v.4.2 (Raymond & Rousset, 1995; Rousset, 2008) was used to test each locus per location for departure from Hardy–Weinberg equilibrium (HWE). The same software was used to assess linkage disequilibrium (LD) between different combinations of paired loci. The analysis of HWE was conducted with a dememorisation period of 10,000 generations, 100 batches and 5,000 iterations per batch. In the case of LD, the number of batches was increased to 1,000. Additionally, the software Micro-Checker v.2.2.3 (Van Oosterhout, Weetman & Hutchinson, 2006) was used to test for systematic distortion of HWE in each locus, which is an indication for the presence of null alleles, large allele dropout or other scoring errors. Sequential Bonferroni corrections for multiple comparisons were used to adjust the threshold of statistical significance in both analyses (Holm, 1979; Rice, 1989). Loci that departed from HWE, showed LD or evidence of errors in scoring were not included in subsequent analyses.

Genetic diversity within each locality was determined through the estimation of number of alleles per locus and locality, gene diversity, observed and expected heterozygosity and allelic richness using the software ARLEQUIN v.3.5.1.2 (Excoffier & Lischer, 2010) and GENEPOP v.4.2 (Raymond & Rousset, 1995; Rousset, 2008). The permutation of localities (1,000 randomizations) was used to determine differences in genetic diversity using the software FSTAT v.2.9.3.2 (Goudet, 2001).

A hierarchical analysis of molecular variance (AMOVA) loci by loci was carried out in ARLEQUIN v.3.5.1.2 (Excoffier, Smouse & Quattro, 1992; Excoffier & Lischer, 2010). AMOVA (with 20,000 permutations) was used to determine genetic diversity (as a source of covariance) and its significance within and between localities, and between islands. AMOVA was also performed by grouping islands according to the connectivity predicted by oceanic currents and assuming passive larval dispersal (Treml et al., 2008), and by grouping them based on clusters obtained from the program STRUCTURE (see below). The purpose of this last analysis was to evaluate the strength of the separation between the inferred clusters. Population pairwise ${F}_{\mathrm{ST}}$ values were estimated and significance was assessed using 20,000 permutations, False Discovery Rate (FDR, Benjamini & Hochberg, 1995), and Bonferroni correction. Confidence intervals were estimated with the package *diveRsity* (Keenan et al., 2013).

The estimation of the number of distinct populations and the assignment of individual samples to populations was done using the software STRUCTURE v.2.3.4 (Falush, Stephens & Pritchard, 2003; Falush, Stephens & Pritchard, 2007; Hubisz et al., 2009; Pritchard, Stephens & Donnelly, 2000). The number of potential populations or clusters (*K*) was evaluated using values for *K* from 1 to 10, with at least 12 independent runs for each value. Uniform priors in an admixture ancestry model were used in each run with a burn-in period of 200,000 generations, a posterior sampling chain of 1,000,000 generations and the assumption of correlated allele frequencies among samples. The determination of the most accurate value for *K* was evaluated using the statistic Δ*K* following the methodology of Evanno, Regnaut & Goudet (2005).

Finally, genetic structure and differentiation between localities was also determined by a discriminant analysis of principal components (DAPC; Jombart, Devillard & Balloux, 2010). This last analysis was performed using the package *adegenet* (Jombart, Devillard & Balloux, 2010) implemented in R v.3.0.1 (http://www.r-project.org/). Individuals with missing data were not included in this analysis.

## Results

### Hardy–Weinberg equilibrium, linkage disequilibrium and possible genotyping errors

Based on an exact test using one Markov chain for each locus per sampled location, three loci deviated significantly from HWE in almost all locations (initial *p*-value < 0.05 after sequential Bonferroni correction). These loci were *Tama01, Maki11* and *Tama11*, which deviated in more than 8 (out of 14) locations. Additionally, the analysis with Micro-Checker showed a consistent excess of homozygotes in the same markers, suggesting the presence of null alleles, polymerase stuttering or large allele dropout. Because these results were consistently biased for most of the sampled localities, those three loci were not included in subsequent analyses.

The remaining markers showed HWE, with punctual deviations in some populations (i.e., *Yukina05, Mak03, Aya2* and *AyU03* in non-HWE in 2, 1, 3 and 4 localities, respectively). In these cases the tests used did not show evidence of linkage disequilibrium, null alleles, or biases in the identification of genotypes. Thus, these loci were included in all subsequent analyses.

### Gene diversity within populations

Most of the microsatellite loci were highly polymorphic. Two exceptions were found: *Yukina08* in Johnston Atoll and *Maki12* in Moorea and Johnston Atoll, where only one allele was fixed in the populations. These two markers, as well as *Aya2*, showed the lowest number of alleles (6–8) for the entire set of samples and the lowest number of alleles per location (1–6) (Table 1 and Table S2). On the other hand, the rest of the markers were highly polymorphic with a total number of alleles ranging between 9 and 18, with 2–14 alleles per locus per locality.

The high genetic diversity suggested by the number of alleles in each location contrasted with the findings on allelic richness. The high genetic variability within localities precluded observing differences in allelic richness between localities (Table S2). After correction for different sampling sizes using a rarefaction analysis, the lowest value of allelic richness was found at Johnston Atoll (2.85), the highest values were observed for the localities in Guam (around 5.11), Japan (5.20) and the GBR (5.22). Despite these differences, the allelic accumulation function, which predicts the expected number of alleles to be observed if the localities would have had the same sample size (Van Loon, Cleary & Fauvelot, 2007), did not show differences in allelic richness between localities due to a broad confidence interval (Fig. S1).

**Table 2:** Pairwise ${F}_{\mathrm{ST}}$ values for 13 *Acanthaster ‘planci’* localities. Bold numbers and gray cells indicate statistical significance after FDR and Bonferroni correction at *P* < 0.05. The red and blue squares show the regional group found in this study based on STRUCTURE and PCA analyses (see text for details).

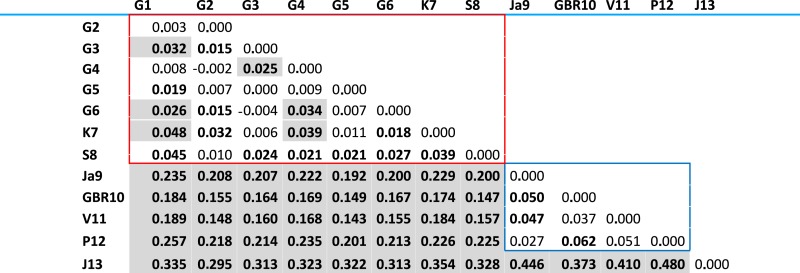

### Genetic structure

Pairwise ${F}_{\mathrm{ST}}$ values obtained ranged from 0.000 to 0.480 (Table 2). In general, the lowest values were found between localities inside Guam and the highest in most comparisons involving Johnston Atoll. ${F}_{\mathrm{ST}}$ analysis showed three significantly differentiated groups: (1) all the localities immediately around Guam, as well as Kingman and Swains Island (2) Japan, Philippines, GBR, and Vanuatu; and (3) Johnston Atoll, which was significantly different from all other localities. Pairwise ${F}_{\mathrm{ST}}$ values between localities inside regional groups were significantly lower (around 0.05 as the highest value) than between localities from different regional groups (${F}_{\mathrm{ST}}$ values higher than 0.15) (Table 2, confidence intervals in Table S3). Moorea was significantly different from the other localities, showing the lowest pairwise ${F}_{\mathrm{ST}}$ with Vanuatu (0.203) and an overall average value of 0.260. However, due to the wide confidence intervals for the ${F}_{\mathrm{ST}}$ values (see Table S3) obtained from comparisons involving Moorea, likely resulting from the low number of samples available from this locality (*N* = 5), the genetic similarity of Moorea with other localities cannot be precisely assessed and the status of Moorea as significantly different from all other localities must be taken with caution. Thus, to avoid artifacts, Moorea was not segregated as fourth group and was not included in some subsequent analyses (e.g., DAPC).

Within the first group, it is important to note that all the localities around Guam, except Tipalao Bay (G1), had statistically significant genetic similarities with Kingman Reef and/or Swains Island. This is noteworthy because the population in Tipalao Bay was the only aggregation in the southwest coast of Guam during surveys and COTS were almost absent in adjacent reefs (C Caballes, pers. obs., 2006). Moreover, the ${F}_{\mathrm{ST}}$ values obtained for several of the comparisons between Guam populations were higher than those obtained in comparisons between Kingman or Swains and localities around Guam. For example, the comparison between North Haputo point (G4) and Tagachan point (G6), and Tipalao Bay (G1) and Taguan Point (G3) resulted in ${F}_{\mathrm{ST}}$ values of 0.034 and 0.032, respectively. In contrast, the ${F}_{\mathrm{ST}}$ between Kingman Island (K) and Taguan Point (G3) in Guam was 0.006 and between Swains Island (S) and Uruno Point (G2) was 0.010. However, the genetic differences observed among localities around Guam and between Guam and Kingman or Swains Island were not statistically significant due to the broad confidence intervals for the ${F}_{\mathrm{ST}}$ values.

According to the results of the AMOVA, the percentage of genetic covariance explained by individual variation was 84% (variation within localities in Table 3); while 15% of the genetic variation can be attributed to differentiation between islands and only 1% is explained by variation among localities (i.e., sampling sites). The same analysis grouping islands according to the three groups found in the pairwise ${F}_{\mathrm{ST}}$ analysis and in Bayesian clustering with STRUCTURE (see below) resulted in a reduction in the percentage of genetic variance explained by individual variation (Variation within localities = 80% in case 4 and 5 from Table 3) and in an increase in the percentage of variance explained by regions (18%). Grouping islands based on oceanic currents (Treml et al., 2008) also increased the variance explained by regions (14% and 17% in case 2 and 3 from Table 3) and ${F}_{\mathrm{ST}}$ values, but the values were lower than in the previous two analyses (case 4 and 5 from Table 3).

**Table 3:** Results from AMOVA grouping localities by islands and regional groups. In all cases *p*-values were highly significant (<0.001 after Bonferroni and FDR correction). In all cases, all localities were included in the analysis, but the regional groups change. Groups in case 2 and 3 are based on models using oceanic currents and different PLDs (30 and 60 days) (Treml et al., 2008). Groups in case 4 and 5 are based on findings from Bayesian analyses. Names of localities as in Table 1.

Factor	Islands	Localities within islands/regions	Within localities	Total
1. Grouping by islands				
SS	216.80	29.47	1790.13	2036.39
VC	0.62	0.04	3.48	4.14
PV	15.02	1.03	83.95	100.00
${F}_{\mathrm{ST}}$				0.160
2. [GBR + V]				
SS	208.16	38.11	1790.13	2036.39
VC	0.61	0.06	3.48	4.14
PV	14.65	1.35	84.00	100.00
${F}_{\mathrm{ST}}$				0.160
3. [GBR + V + S + K + Guam]				
SS	127.67	118.60	1790.13	2036.39
VC	0.77	0.22	3.48	4.46
PV	17.17	4.84	77.99	100.00
${F}_{\mathrm{ST}}$				0.220
4. STRUCTURE groups: [Guam + K + S] + [Ja + GBR + V + P]			
SS	175.03	71.24	1790.13	2036.39
VC	0.80	0.09	3.48	4.36
PV	18.34	1.97	79.69	100.00
${F}_{\mathrm{ST}}$				0.203
5. STRUCTURE group 1: [Guam + K + S]				
SS	201.32	44.95	1790.13	2036.39
VC	0.79	0.06	3.48	4.33
PV	18.34	1.38	80.28	100.00
${F}_{\mathrm{ST}}$				0.197

**Notes.**

SS sum of squares VC variance component PV percentage of covariance

### Population structure

**Figure 2:**
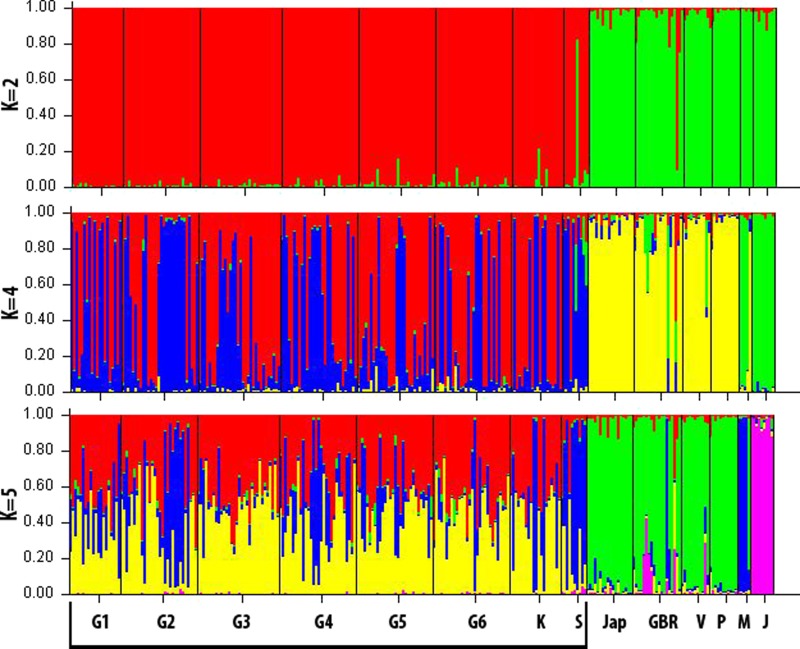
Graphical summary of Bayesian clustering results. Samples were assigned among 2, 4 and 5 genetic clusters (*K*). Each colour represents the probability of corresponding to a specific cluster. Each locality is separated by a black line. The Guam group (Guam, Kingman and Swains Islands) is highlighted with the horizontal black line at the bottom.

The Bayesian clustering analysis with STRUCTURE and the estimation of Δ*K* (Evanno, Regnaut & Goudet, 2005) indicated the existence of two peaks in the most likely number of ancestral gene pools. The highest value of Δ*K* was obtained for *K* = 2. A second peak was observed at *K* = 4. This last value corresponds with the point where a significant change in the slope of the likelihood distribution is observed. Moreover, DAPC showed a significantly low Bayesian Information Criterion (BIC) value for the existence of 5 genetically different groups (Fig. 2 and Fig. S2).

When samples were assigned to two genetic groups (*K* = 2), Guam, Kingman Reef and Swains Island clustered together and were significantly different from all other locations sampled in this study (Fig. 2). When the number of genetic groups was increased to four and five, the initial differentiation in two groups was maintained with additional information within each group. First, mixing between Guam, Kingman and Swains was evident, with a different proportion of individuals belonging to each predicted population inside localities (represented as blue and red colours in the bar plot with *K* = 4, and blue, red and yellow in the plot with *K* = 5). In the second group, the change in the number of predicted populations from 2 to 5 revealed the differentiation of islands such as Moorea and Johnston Atoll, and increased the heterogeneity observed inside the GBR.

### Differentiation and relatedness between localities

**Figure 3:**
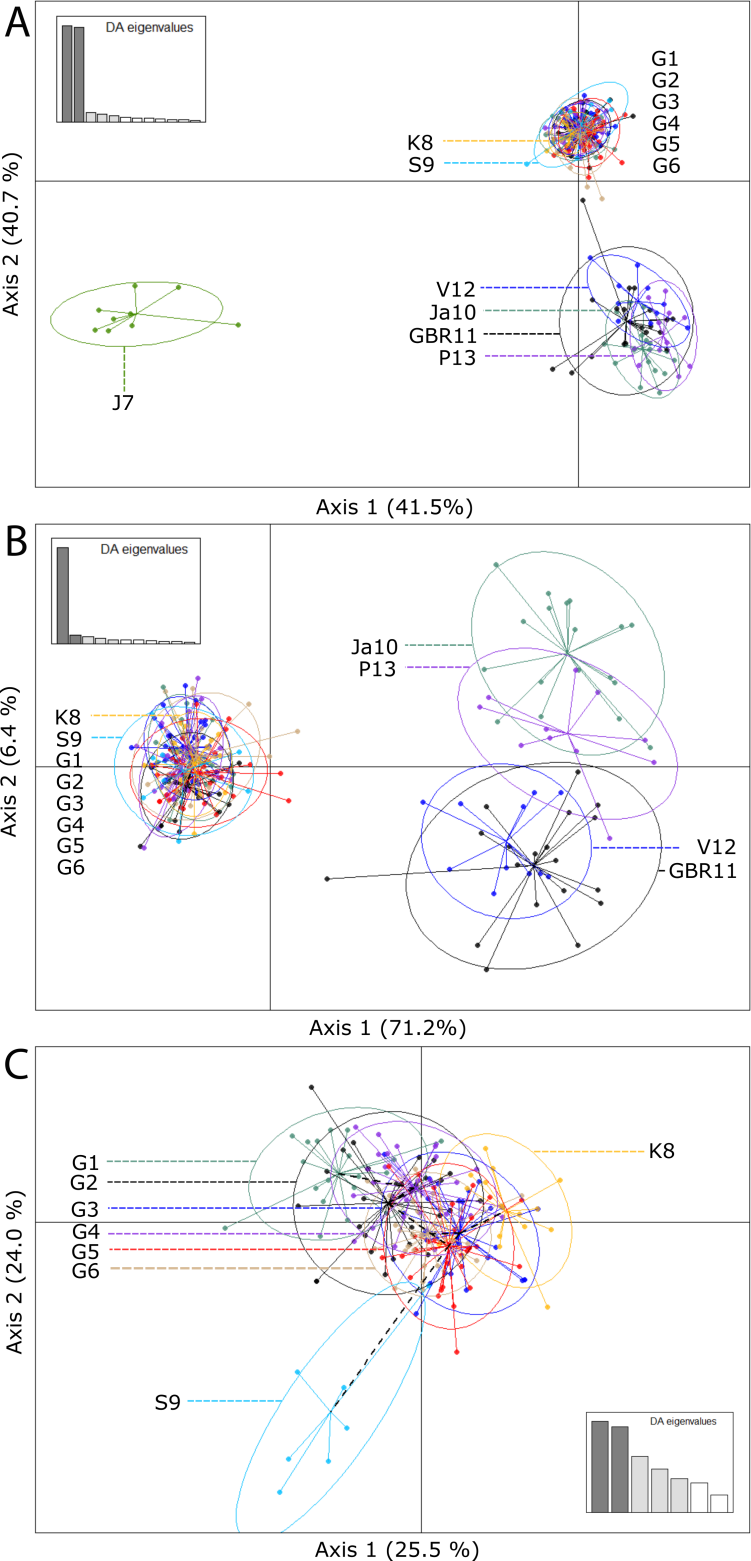
Scatterplots of the discriminant analysis of principal components (DAPC) for all localities (A), group 1 (Guam, Kingman and Swains Islands) and 2 (GBR, Japan, Philippines, Vanuatu) (B) and only the group 1 (C). Individual genotypes appear as dots surrounded by 95% inertia ellipses. Eigenvalues show the amount of genetic information contained in each successive principal component with *x*- and *y*-axes constituting the first two principal components, respectively.

The first two components of the DAPC explained 41.5% and 40.7% of the variation in the dataset and these results were consistent with the results found with STRUCTURE (Fig. 3A). In this analysis, the level of genetic similarity was represented by a clustering of the genotypes by locality. When all localities sampled were included in the analysis, Johnston Atoll stood out as a strongly divergent group, possibly isolated and without gene flow to/from the two main locality groups.

Because the genetic differentiation of Johnston Atoll with the other islands was strong, additional DAPC analyses were performed (Figs. 3B and 3C). Aiming to gain insights into the genetic differentiation within each group, in one analysis Johnston Atoll was excluded and in a second one only localities from Guam, Kingman and Swains Island were included. Excluding Johnston Atoll, the level of clustering between localities was more pronounced for the first group of localities (Guam, Kingman and Swains) than for the second group (GBR, Japan, Philippines and Vanuatu). This is of considerable interest, because geographic distance between Guam, Kingman, and Swains was higher compared to distances between localities in the second group. Additionally, although gene flow between all the locations of the second group appears likely, there was an apparent subdivision inside this group with the GBR grouping with Vanuatu, and Japan grouping together with the Philippines (Fig. 3B). The differentiation between these two subgroups was given only by the second axis (*Y* axis) of the DAPC and the percentage of variance explained by this axis was low (6.4% of the variance).

Localities within Guam (Fig. 3C) showed higher levels of genetic similarity. However, it is important to observe that this genetic similarity is variable, with some localities in Guam more related to Kingman (for example G3, G4, G5 and G6) and with a slight overlap with Swains and other localities more isolated from Kingman and Swains (locality G1). These results suggest the existence of genotypes in Guam that are different to those found in Kingman and Swains Islands (unique haplotypes).

## Discussion

### Contemporary long-distance dispersal across the Pacific

This study found genetic structure within large geographical regions in the Pacific but also suggests that gene-flow between distant locations likely occurs in *A. ‘planci.’* Given the broad geographical distances separating the localities evaluated here, it is likely that this dispersal occurs in a stepping stone model involving intermediate localities not sampled in this study. The sampled localities in the Pacific were found to be structured in at least three large groups with apparently limited larval dispersal between them. The first group comprised Guam, Kingman Reef and Swains Islands; the second group included the Japan, Philippines, GBR and Vanuatu; and Johnston Atoll was isolated in a third group. Although a general high genetic diversity was found inside each sampled island, our analyses showed strong genetic similarities between localities in Guam, Kingman and Swains Island suggesting larval dispersal between these geographically distant regions. Gene-flow between these localities in the Pacific Ocean has been also inferred using allozymes from other marine organisms with high larval dispersal potential (e.g., *Linckia laevigata*, Williams & Benzie, 1997; some species of corals, Ayre & Hughes, 2000; sea cucumbers, Uthicke & Benzie, 2003).

Connectivity of marine organisms has been modelled as a diffusion process in which larvae and juveniles are transported by oceanic currents between suitable habitats (Treml et al., 2008; Kool et al., 2011; Treml et al., 2012; Wood et al., 2014; Treml et al., 2015). In these models, one of the criteria determining the connectivity between localities is the duration of the larval stage (planktonic larval duration, PLD)—assuming that organisms with longer PLDs are capable of migrating longer distances. According to these models, the dispersal potential of *A. ‘planci,’* with a PLD of up to 42 days (reviewed in Pratchett et al., 2014), would allow the migration of individuals across long distances (see also Vogler et al., 2013), potentially connecting the islands of Guam, Kingman and Swains in a stepping-stone model. Our results from microsatellite data are consistent, in part, with this model as evidenced by the lack of genetic structure between Guam and geographically distant COTS samples from Kingman Reef and Swains Island. However, intermediate islands/reefs between Guam and Kingman/Swains (e.g., Marshall Islands, Pohnpei) need to be sampled in the future to test the stepping-stone model proposed here.

Long distance connectivity is especially important during events like *El Niño* (Treml et al., 2008), when some current systems in the Pacific deviate from established patterns. Treml et al. (2008) suggested that for corals, Pacific-wide connectivity is strongly reduced when a probability of successful dispersal of 0.5 is selected. The strong genetic differentiation observed between Guam and other Western Pacific localities, namely Philippines and Japan, can be interpreted as a westward drop in larval dispersal, likely caused by the reduced strength of the oceanic currents flowing East-West and the lack of stepping stones between these localities. This combination would make East-West larval dispersal difficult even for organisms with long PLDs (Treml et al., 2008; Kool et al., 2011; Treml et al., 2015).

There are notable discrepancies between our study and previous studies using the control region of the mtDNA (Timmers et al., 2012; Vogler et al., 2013) in terms of population structure and differentiation between localities in the Pacific Ocean. According to Timmers et al. (2012), populations in the Central Pacific are genetically differentiated into three main regions with few or no shared haplotypes: North, South and North-West Pacific. They found that the Johnston Atoll is part of the North region; Kingman, Swains and Moorea Islands belonged to the South region and Guam to the North-West region. Additionally, Vogler et al. (2013) grouped Guam in a large western region with Japan, Philippines and the GBR. Our results, using microsatellites, agree with these results in the broad geographical zonation in the Pacific, but this study also evidenced strong genetic similarity between supposedly distinct regions mentioned above, suggesting possible larval dispersal and gene flow between long distance localities in the south-central Pacific and the north-west Pacific regions, such as Guam, Kingman and Swains Islands.

Discordances between mtDNA and microsatellites data had been previously reported for *A. ‘planci’* populations from the Pacific Ocean. Using microsatellites, Yasuda et al. (2009) found that *A. ‘planci’* populations in the western Pacific (i.e., Japan and Philippines), the GBR, and the North Pacific Islands (i.e., Palau, Majuro and Pohnpei, which are geographically close to Guam) belonged to different genetic groups. In contrast, when using mtDNA control region, Vogler et al. (2013) found that *A. ‘planci’* samples from Palau were closer to the western Pacific population, while Majuro and Pohnpei were more related to the GBR.

Patterns of low control region mitochondrial divergence in Pacific COTS have been interpreted as a result of occasional exchange of larvae between distant areas, the retention of ancestral polymorphism or a signature of ancient gene flow (Timmers et al., 2012). In addition, Vogler et al. (2013) found signatures of a recent population expansion in a large group of Pacific populations (including Guam). Our analysis suggests that the mitochondrial divergence detected between some distant localities may gradually erode due to the likely existence of more contemporary gene flow between these localities. We would like to note that in this study, the direction, frequency and magnitude of the gene flow could not be assessed, and that this will require more extensive sampling of intermediate localities. Other explanations for the discrepancy between control region mtDNA and microsatellite data exist and include the non-neutral evolution of mtDNA (Ballard & Whitlock, 2004) with the potential for sex-biased migration or selection on specific haplotypes; differences in the effective population size, resulting in differences in the effect of genetic drift (Shaw, Arkhipkin & Al-Khairulla, 2004); or the higher likelihood for homoplasy in microsatellites over longer periods of time, due to higher mutation rates (O’Reilly et al., 2004). A more dense spatial sampling would allow to better understand which processes are involved in the mito-nuclear discordance observed.

### Differences within Guam

Despite the apparent lack of genetic structure between some geographically distant regions, some evidence of genetic differentiation among localities around Guam were found. For example, it was found that some localities around Guam (e.g., Tipalao Bay (G1), Uruno Point (G2) and North Haputo Point (G4)) were genetically differentiated from Kingman Reef and/or Swains Islands (considering pairwise ${F}_{\mathrm{ST}}$ values), but other localities around Guam (e.g., Taguan (G3) or Tanguisson (G5)) are suggesting dispersal from and to these distant localities. A similar pattern was found in the discriminant analysis of principal components (DAPC, Fig. 3); however, these genetic differences were not statistically significant due to the large confidence intervals of the ${F}_{\mathrm{ST}}$ values. Additional studies are needed to determine if those differences are biologically relevant or result from an increased variance inherent to the implemented methodology. The exact factors causing the observed differentiation remain to be determined, especially considering that previous studies using other markers, such as allozymes or control region mtDNA, have also identified genetic differences between local COTS populations (Benzie, 1999a; Benzie & Stoddart, 1992; Nash, Goddard & Lucas, 1988; Nishida & Lucas, 1988; see Timmers et al., 2012 for within island differentiation).

The genetic structure of COTS populations can be affected by a number of factors, such as different oceanographic conditions, climatic fluctuations, local adaptation and differential mortality of pre-settlement larval stages (Benzie & Stoddart, 1992; Yasuda et al., 2009). In addition, the distribution and dynamics of *A. ‘planci’* populations are sensitive to changes in food availability (abundance of coral prey), food quality (preferred coral species), and population densities (De’ath & Moran, 1998; Kayal et al., 2012). The sampled coastal localities in Guam differ in terms of the amounts of riverine discharge and hydrodynamic patterns (Wolanski et al., 2003), and the reefs vary in coral cover and community structure (Burdick et al., 2008). Changes in local conditions linked to anthropogenic activities (e.g., increased sedimentation, terrestrial runoff and overfishing; Brodie et al., 2005) are capable of triggering primary outbreaks and may also facilitate larval survival and settlement success, leading to increased adult numbers and secondary outbreaks. All these risk factors have increased magnitude and frequency during the last decades in Guam (Burdick et al., 2008; Gawel, 1999) and may explain the increase in the frequency and impact of *A. ‘planci’* in this island and the structuring of its populations. Further studies are warranted to assess the relative importance of these local environmental factors on the genetic structure of COTS populations.

## Conclusions

In this study, the genetic structure of the crown-of-thorns seastar (*Acanthaster ‘planci’*) around Guam was evaluated using microsatellites and compared to spatially distinct localities in the Pacific. Genetic structure was detected within the sampled Pacific localities, which suggests clustering of reefs into broad geographic groups, some of them consistent with previous findings based on the control region of the mtDNA. A lack of genetic structure was suggested between Guam Island and distant reefs, such as Kingman and Swains, previously considered isolated regions. Additional studies including a denser spatial sampling are needed to test the strength and direction of putative gene flow between these localities and whether such putative long-distance dispersal events have an impact at the local demographic level.

## Supplemental Information

10.7717/peerj.1970/supp-1 Figure S1 Predicted allelic richness in function of sample size Each line represents sampled localities. Localities with the lowest and highest allelic richness are shown in red and blue respectively, with their variance in dash lines.

10.7717/peerj.1970/supp-2 Figure S2 Results of the discriminant analysis of principal components (DAPC) Values of the Bayesian Information Criteria (BIC) in function of number of clusters.

10.7717/peerj.1970/supp-3 Table S1 Raw genotype data per locus and individual Each line corresponds to one individual. The values are given in length (bp) of the fragment. Empty spaces are missing data.

10.7717/peerj.1970/supp-4 Table S2 Genetic diversity estimates for 10 microsatellite loci all the localities N, number of individuals; Na, number of allele per locus; Ar, allelic richness; Ho, observed heterozygosity; He, expected heterozygosity; FIS, inbreeding coefficient; S.D., standard deviation.

10.7717/peerj.1970/supp-5 Table S3 Pairwise F[i]_ST bootstrap CI, confidence intervals; BC, Bonferroni correction; FDR, False discovery rate.

## Additional Information and Declarations

### Data Availability

The following information was supplied regarding data availability: The raw genotype data is provided as Table S1.
