# Supplementary figures and images for: Genetic structure of the crown-of-thorns seastar in the Pacific Ocean, with focus on Guam
Source: PeerJ. 2016 May 5;4:e1970. doi: 10.7717/peerj.1970 (PMC4860296; doi:10.7717/peerj.1970)

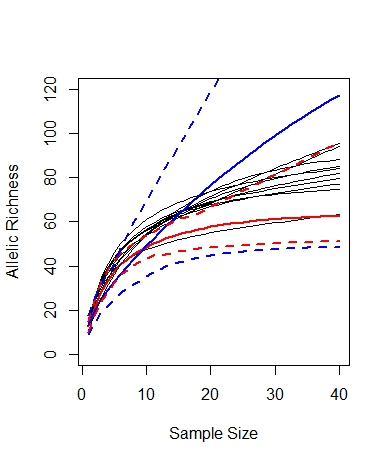

Supplement: Figure S1 — Each line represents sampled localities. Localities with the lowest and highest allelic richness are shown in red and blue respectively, with their variance in dash lines. [file peerj-04-1970-s001.jpeg]

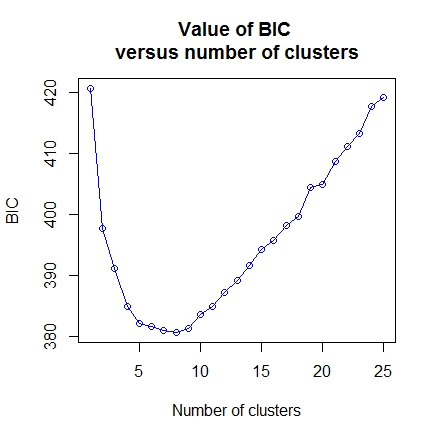

Supplement: Figure S2 — Values of the Bayesian Information Criteria (BIC) in function of number of clusters. [file peerj-04-1970-s002.jpeg]
